# Asymptotic Pomeranchuk instability of Fermi liquids in half-filled Landau levels

**DOI:** 10.1038/s41598-023-28614-z

**Published:** 2023-01-25

**Authors:** Jorge Quintanilla, Orion Ciftja

**Affiliations:** 1 Physics of Quantum Materials Research Group, School of Physics and Astronomy, University of Kent, Canterbury, CT2 7NH UK; 2 ISIS Facility, STFC Rutherford Appleton Laboratory, Harwell Science and Innovation Campus, Didcot, OX11 0QX UK; 3 Department of Physics, Prairie View A &M University, Prairie View, TX 77446 USA

**Keywords:** Materials science, Physics

## Abstract

We present a theory of spontaneous Fermi surface deformations for half-filled Landau levels (filling factors of the form $\nu =2 \, n+1/2$). We assume the half-filled level to be in a compressible, Fermi liquid state with a circular Fermi surface. The Landau level projection is incorporated via a modified effective electron-electron interaction and the resulting band structure is described within the Hartree-Fock approximation. We regulate the infrared divergences in the theory and probe the intrinsic tendency of the Fermi surface to deform through Pomeranchuk instabilities. We find that the corresponding susceptibility never diverges, though the system is asymptotically unstable in the $n \rightarrow \infty$ limit.

## Introduction

The discoveries of the integer quantum Hall effect (IQHE)^1^ and the fractional quantum Hall effect (FQHE)^2^ have stimulated many studies on the properties of two-dimensional (2D) strongly correlated electronic systems in a perpendicular magnetic field. New ideas such as existence of fractionally charged quasiparticles^3^ or the prediction of composite fermions^4^ have broadened our understanding of nature and have profoundly affected physics and other sciences. Theories built on these ideas have also proven very reliable to explain unusual properties of strongly correlated electronic systems in the extreme quantum Hall regime at various filling factors.

The basic theory of the quantum Hall phemonena can be understood within the framework of the theory of Landau states. The quantum energies of a 2D system of electrons in a perpendicular magnetic field become quantized in highly degenerate discrete Landau level-s (LL-s). The degeneracy of each LL is proportional to the magnitude of the magnetic field. The energy difference between two successive LLs is $\hbar \omega _{c}$ where $\omega _c$ is the cyclotron frequency and $\hbar$ is the reduced Planck’s constant. A large magnetic field leads to both a large number of degenerate quantum states for each LL as well as larger energy separation between any two adjacent energy levels. For these conditions, a general assumption is to consider the electrons also spin-polarized and, thus, neglect spin effects. The most important parameter to characterize the state of the system of electrons in the quantum Hall regime is the so-called filling factor, $\nu$ defined as the ratio $N/N_s$ where *N* is the number of electrons and $N_s$ is the degeneracy of a given LL. The IQHE happens for filling factors of the form, $\nu =1, 2, \ldots$. On the other hand the FQHE occurs when at least one LL is partially filled. The most famous FQHE states are those corresponding to an odd-denominator filling factor $\nu =1/3$ and 1/5. Such states are described very well by Laughlin’s theory^3^ and, for this reason, are routinely called Laughlin’s states.

Somewhat more poorly-understood are the half-filled states that range from composite fermions that may undergo a pairing transition in a half-filled LL^5^ to anisotropic states in high LL-s^6^. In particular, the issue of anisotropy was addressed theoretically quite early on^7,8^. Moessner and Chalker^8^ showed using Hartree-Fock theory that a striped charge density wave (CDW) prevails in the limit of very high LL-s if the interaction is of sufficiently short range relative to the magnetic length. In fact ample theoretical evidence of their existence has accumulated^9,10^. While a stripe CDW phase is sufficient to explain anisotropy, other, competing structures may lead to an anisotropic response as well. Indeed it has been pointed out^8^ that, when the range of the effective interaction is comparable to the magnetic length, uniform states may win over the striped phase and, a few years later, it was shown^11^ that melting of the stripes could lead to a nematic phase. In this state, translational symmetry is completely restored but the system remains anisotropic. Interestingly such smectic and nematic states can be regarded as the ‘missing links’ between the Wigner crystal and Fermi liquid states, based on a proposed general picture of strong correlations^12^.

In order to explain the emergence of anisotropy in Fermi liquid states at filling factor $\nu =2 n+1/2$ $(n \ge 2)$, there has been a surge of interest in the Pomeranchuk instability (PI)^13^. Through such mechanism, a compressible Fermi liquid state (presumably the half-filled states in high LL-s) may “spontaneosly” enter into an anisotropic nematic state characterized by a deformed Fermi surface. In this scenario anisotropy emerges at one-particle level. In fact, the wave function for the nematic state proposed by Oganesyan et al.^14^ consists of single-particle 2D plane-wave states that form an elliptical Fermi sea^15^. It is also plausible to suggest that anisotropy may emerge at a two-particle level, too. In this second scenario, one would start with a broken rotational symmetry wave function that contains a suitable symmetry-breaking parameter in the two-particle correlation part of the wave function and a Slater determinant of 2D plane waves that form a standard circular Fermi sea^16,17^.

In this work we test the first of the above two scenarios. Specifically, we address the question of formation of a nematic state (or its higher angular momentum generalisations) from the opposite direction in the phase diagram of Fradkin et al.^12^, i.e. from the Fermi liquid side. Assuming a circular Fermi liquid ground state as a starting point, our *modus operandi* is to derive an effective electron-electron interaction that takes into account the projection onto the relevant half-filled LL. We then investigate whether this circular Fermi surface is unstable to small, point group symmetry-breaking deformations that are the 2D counterpart of the three-dimensional (3D) PI phenomenon^13^.

## Results and discussions

### Model and theory

The interaction of a charged particle with a magnetic field leads to many interesting quantum phenomena. The standard model for a system of electrons in the quantum Hall regime assumes that electrons are confined to an ideal 2D layer. Such a system can be experimentally created at the interface between semiconductor and insulator or between semiconductors. A typical device is the *GaAs*/*AlGaAs* heterostructure. The 2D system of electrons is subjected to a uniform magnetic field perpendicular to the 2D layer. The vector potential characterizing the magnetic field, $\vec {B}=\vec {\nabla } \times \vec {A}(\vec {r})$ can be conveniently written in a symmetric gauge as $\vec {A}(\vec {r})= \frac{1}{2} \left( \vec {B} \times \vec {r} \right)$ where $\vec {B}$ is the magnetic field and $\vec {r}$ is the position vector of the electrons. Generally speaking, charged elementary particles such as electrons also possess a quantum spin. There is a straightforward way to describe the interaction of the electron’s spin with a magnetic field. This interaction gives rise to the so-called Zeeman spin energy term. However, to reveal the essence of the quantum Hall system, it is a common practice to consider a spinless theory by ignoring the spin degree of freedom. This approach represents a good approximation to the spin-frozen system, where the Zeeman energy is large and all spins are frozen into their polarized states. Therefore, from now on, we ignore all spin effects and focus only on the spatial motion of electrons in a magnetic field. The part of the quantum Hamiltonian which adequately describes the spatial motion of a charged particle in a magnetic field is given by the kinetic energy operator, ${\hat{K}}$ written as:

1 $$\begin{aligned} {\hat{K}}=\frac{1}{2 \, m_{e}} \sum _{i=1}^{N} \left[ \hat{\vec {p}}_i -q \, \vec {A}(\vec {r}_i) \right] ^2 \ , \end{aligned}$$

where $\hat{\vec {p}}=({\hat{p}}_x,{\hat{p}}_y)$ is the 2D linear momentum operator, $m_e$ is the (effective) mass of electrons and $q=-e$ $(e>0)$ is electron’s charge. The strong magnetic field quantizes the electrons’s motion on the plane and quenches the kinetic energy of each electron to a discrete set of LL-s separated by the relatively large cyclotron energy, $\hbar \, \omega _c =\hbar \, e \, B/m_e$. The ground state energy of the system is commonly referred to as the lowest Landau level (LLL). It is customary (or simply implied) in many quantum Hall studies to assume that the direction of the perpendicular magnetic field is given as $\vec {B}=(0, 0, -B)$. The choice of the negative sign of $\vec {B}$ is a matter of convenience, allowing one to express the resulting LLL wave functions of the electrons in terms of mathematical expressions that depend on the complex variable $z=x+i y$ where $i=\sqrt{-1}$ is the imaginary unit. A detailed solution of the problem of Landau states in a symmetric gauge is readily available from the literature^18^.

The charge neutrality holds in any system, and in a quantum Hall system it is guaranteed by the positive charge of a neutralizing background. The appropriate choice of the geometry of the background is a disk for a symmetric gauge choice of the vector potential. Therefore, in a disk geometry model, we assume that the electrons are immersed in a uniform positively charged finite disk of area $\Omega _N=\pi \, R_N^2$ where $R_N$ is the radius of the disk. The density of the system (number of electrons per unit area) or otherwise the uniform density of the background, $\rho _0=N/\Omega _N$, is constant. The uniform electron density of the system can also be written as: $\rho _0=\nu /(2 \, \pi \, l_0^2)$, where $l_0=\sqrt{\hbar /(e \, B)}$ is the electronic magnetic length. From here, one can identify the value of the radius of the disk for any given $\nu$ and *N*. The total quantum Hamiltonian of the system consists of the kinetic energy term ${\hat{K}}$ and a potential energy term ${\hat{V}}$ (the Zeeman term is not included):

2 $$\begin{aligned} {\hat{H}}={\hat{K}}+{\hat{V}} \ . \end{aligned}$$

The potential energy operator:

3 $$\begin{aligned} {\hat{V}}={\hat{V}}_{ee}+{\hat{V}}_{eb}+{\hat{V}}_{bb} \ , \end{aligned}$$

consists of electron-electron (ee), electron-background (eb) and background-background (bb) interaction potentials written as:

4 $$\begin{aligned} {\hat{V}}_{ee}=\sum _{i<j}^{N} v(\vec {r}_i-\vec {r}_j) \ , \end{aligned}$$

5 $$\begin{aligned} {\hat{V}}_{eb}= -\rho _0 \sum _{i=1}^{N} \int _{\Omega _N} d^2r \, v(\vec {r}_i-\vec {r} ) \ , \end{aligned}$$

and

6 $$\begin{aligned} {\hat{V}}_{bb}=\frac{\rho _0^2}{2} \int _{\Omega _N} d^2r \int _{\Omega _N} d^2r^{\, \prime } \ v(\vec {r}-\vec {r}^{\, \prime }) \ , \end{aligned}$$

where

7 $$\begin{aligned} v(\vec {r}_i-\vec {r}_j)=\frac{e^2}{4 \, \pi \, \epsilon \, |\vec {r}_i-\vec {r}_j|} \ , \end{aligned}$$

is the Coulomb interaction potential. For simplicity we will take the dielectric function $\epsilon$ to be a constant. In all above expressions, $\vec {r}_{i}$ (or $\vec {r}_{j}$) denote electronic 2D position vectors, while $\vec {r}$ and $\vec {r}^{\, \prime }$ are background coordinates. Each of the *N* position variables of electrons, $\{ \vec {r}_i \}$ extends all over space ($-\infty$ to $+\infty$). However, background coordinates, $\vec {r}$ ( or $\vec {r}^{\, \prime }$) are confined within the finite disk, namely $0 \le |\vec {r}| \le R_N$ (or $0 \le | \vec {r}^{\, \prime } | \le R_N$). The model described and its Hamiltonian are standard for a quantum Hall state in a disk geometry. Further details of the notation and formalism adopted can be found, for instance, in the work by Ciftja and Wexler^19^ that discusses the application of the Monte Carlo simulation method for Laughlin-like states in a disk geometry.

### Fermi liquid state at a half-filled Landau level

As was discussed more generally in 3D, for several model effective interactions^20^, key requirements to have a PI are: (i) a sharp feature in the interaction potential at some characteristic distance; and (ii) for this characteristic length to be larger than the separation distance between particles in the system. Indeed the range of the effective interaction potential that we will derive below is longer than the magnetic length. Moreover it has non-monotonic features that develop into a sharp kink at a particular distance that increases as the order of the LL-s increases. On the other hand, the average distance between particles in a half-filled LL is fixed by the mangetic length. Thus both of the above conditions are met asymptotically for sufficiently high LL-s. Testing the ensuing expectation of an intrinsic tendency to a PI for a high half-filled LL is the main purpose of this work.

We consider filling factors of the form: $\nu =2 \, n+ \nu ^{*}$ where $n=0, 1, \ldots$ is the index of the uppermost half-filled LL ($\nu ^{*}=1/2$) assumed to be fully spin-polarized. According to the Halperin-Lee-Read theory^21^, a 2D electron system in an external magnetic field, at half filling factor, can be transformed to a mathematically equivalent system of fermions interacting with a Chern-Simons gauge field such that the average effective magnetic field acting on the fermions is zero. That theory implies the existence of a well-defined Fermi surface for the fermions of a system with no impurity scattering (ignoring the fluctuations in the gauge field). Various scenarios may occur when gauge fluctuations are taken into account, but subsequent research has so far established that many features of the Fermi surface exist in all these cases. For this reason, a Fermi liquid theory treatment of such a state is a good starting point. Based on these arguments, one may safely assume that the $N^{*}$ electrons of the half-filled LL form a 2D circular Fermi liquid state (with density $\rho ^{*}$) that effectively sees no magnetic field. We have $\nu ^{*} = 2 \, \pi \, l_0^2 \, \rho ^{*}$ where the magnetic length, $l_0$ represents the characteristic length scale in the problem. Since $\nu ^{*}=1/2$ and $\rho ^{*}=\pi \, k_F^2/(2 \, \pi )^2$, the radius of the circular 2D Fermi wave vector is $k_F=1/l_0$.

For such a case study, the Fermi liquid state for $N^{*}$ electrons in the uppermost half-filled LL is described by a Rezayi-Read (RR) wave function^22^ which, in disk geometry, has the form:

8 $$\begin{aligned} \Psi _{n \, , \, \nu ^{*}=1/2}={\hat{P}}_{n} \biggl [ Det \left| e^{i \, \vec {k}_{\alpha } \, \vec {r}_j} \right| \, \Psi _{B \, n} \biggr ] \ , \end{aligned}$$

where $Det \left| e^{i \, \vec {k}_{\alpha } \, \vec {r}_j} \right|$ is a Slater determinant wave function of 2D plane wave states, $\Psi _{B \, n}$ is a Bose Laughlin state appropriate for the *n*-th LL and ${\hat{P}}_{n}$ is a *n*-th LL projection operator. It is assumed that the underlying spin-resolved LL-s are full and considered inert since they are completely frozen out in the strong magnetic field. This implies that for a filling factor of the form: $\nu =2 \, n+\nu ^{*}$, only the $N^{*}$ electrons of the uppermost half-filled LL need to be considered. This means that, for a spin-polarized system like ours, $N^{*}$ electrons occupy the lowest-lying plane wave states labeled by the momenta, $\{ \vec {k}_{\alpha } \}$ up to the value of the Fermi wave number, $k_F$. As an example to illustrate this point let us consider the RR wave function for $\nu =1/2=\nu ^{*}$ $(n=0)$ that would be written as: $\Psi _{n=0 , \nu ^{*}=1/2}={\hat{P}}_{0} \biggl [ Det \left| e^{i \, \vec {k}_{\alpha } \, \vec {r}_j} \right| \, \Psi _{B \, 0} \biggr ]$ where:

9 $$\begin{aligned} \Psi _{B \, 0}=\prod _{j>k}^{N^{*}} (z_j-z_k)^{2} \ \exp {\left( -\sum _{j=1}^{N^{*}} \frac{|z_j|^2}{4 \, l_0^2} \right) } \ , \end{aligned}$$

is the $\nu ^{*}=1/2$ Bose Laughlin wave function^23^ for the $n=0$ LL (in this case $N=N^{*}$). In the above expression, $z_j=x_j+i \, y_j$ is the 2D position coordinate in complex notation. The effect of ${\hat{P}}_{0}$ (or, more generally, ${\hat{P}}_{n}$) is to convert the plane wave states into operators (involving partial derivatives with respect to complex variable, *z*) that act upon the polynomial part of the Bose Laughlin wave function as explained by Girvin and Jach^24^ for the $n=0$ case. The procedure of how to raise any Laughlin or Bose Laughlin wave function from the $n=0$ LL to an appropriate higher LL with $n \ge 1$ is explained by MacDonald^25^. Having projected the wave function onto the *n*-th LL means that the kinetic energy is quenched to the appropriate value of that level, $\langle {\hat{K}} \rangle /N^{*}=(n+1/2) \, \hbar \, \omega _c$.

Projection onto the *n*-th LL by means of ${\hat{P}}_n$ is generally done by calculating a given operator “sandwiched” between the projection operators. Since the main purpose of the whole procedure is to extract some effective interaction potential between the electrons, the quantity that matters is the projected ee interaction potential. One starts with the (not yet projected) quantity for the $N^{*}$ electrons in the partially filled *n*-th LL (the other underlying levels are considered frozen) written as:

10 $$\begin{aligned} {\hat{V}}_{een}=\frac{1}{2} \int d^2r \int d^2r^{\, \prime } \, \Psi _n^{\dag }(\vec {r}) \, \Psi _n(\vec {r}) \, \, v(\vec {r}-\vec {r}^{\, \prime }) \, \Psi _n^{\dag }(\vec {r}^{\, \prime }) \, \Psi _n(\vec {r}^{\, \prime }) \ , \end{aligned}$$

where the field operators consist of states in the *n*-th LL of the form $\Psi _n(\vec {r})=\sum _{m} \langle \vec {r} | n, m \rangle \, {\hat{a}}_{n, m}$ and $\Psi _n^{\dag }(\vec {r})=\sum _{m} \langle n, m | \vec {r} \rangle \, {\hat{a}}^{\dag }_{n, m}$. Recall that such field operators are Fermi ones that satisfy anti-commutator relationships such as $\{ \Psi _{\alpha }(\vec {r}), \Psi _{\beta }^{\dag }(\vec {r}^{\, \prime }) \}= \delta _{\alpha \, \beta } \, \delta (\vec {r}- \vec {r}^{\, \prime })$, $\{ \Psi _{\alpha }(\vec {r}), \Psi _{\beta }^{}(\vec {r}^{\, \prime }) \}= \{ \Psi _{\alpha }^{\dag }(\vec {r}), \Psi _{\beta }^{\dag }(\vec {r}^{\, \prime }) \}=0$ where $\{ {\hat{A}}, {\hat{B}}\}={\hat{A}} \, {\hat{B}}+{\hat{B}} \, {\hat{A}}$ represents an anti-commutator. One rewrites the above equation as:

11 $$\begin{aligned} {\hat{V}}_{een}=\frac{1}{2} \int d^2r \int d^2r^{\, \prime } \rho _{n}(\vec {r}) \, v(\vec {r}-\vec {r}^{\, \prime }) \, \rho _{n}(\vec {r}^{\, \prime }) \ , \end{aligned}$$

where $\rho _n(\vec {r})=\Psi _n^{\dag }(\vec {r}) \, \Psi _n(\vec {r})$ is the electronic density operator in *n*-th LL. One can transform the density operator in 2D Fourier space as $\rho _n(\vec {k})=\int d^2r \, e^{i \, \vec {k} \, \vec {r}} \, \rho _n(\vec {r})$. Thus, the operator can be rewritten in 2D Fourier space as:

12 $$\begin{aligned} {\hat{V}}_{een}=\frac{1}{2} \sum _{\vec {k}} v( \vec {k} ) \, \rho _n(-\vec {k}) \, \rho _n(\vec {k}) \ , \end{aligned}$$

where $v(\vec {k})={2 \pi e^2}/(4\pi \epsilon \, |\vec {k}| )$ is the 2D Fourier transform of the Coulomb interaction potential. Let us denote by $\hat{{\overline{V}}}_{een}$ the projected counterpart (where “overline/bar” implies projection). Note that projection affects the density operators. By following the Hamiltonian theory recipe^26^, the *n*-th LL density operator, when projected, becomes ${\overline{\rho }}_n(\vec {k})=F_n(k) \, {\overline{\rho }}(\vec {k})$ where ${\overline{\rho }}(\vec {k})$ is an “ordinary” projected density operator and $F_n(k)=L_n\left( \frac{k^2 \, l_0^2}{2} \right) \exp \left( -\frac{k^2 \, l_0^2}{4} \right)$ is a form factor with $L_n(x)$-s being Laguerre polynomials. One ends up with:

13 $$\begin{aligned} \hat{{\overline{V}}}_{een}=\frac{1}{2} \sum _{\vec {k}} v(\vec {k}) \, {\overline{\rho }}_n(-\vec {k}) \, {\overline{\rho }}_n(\vec {k})= \frac{1}{2} \sum _{\vec {k}} v(\vec {k}) \, F_{n}(k)^2 \, {\overline{\rho }}(-\vec {k}) \, {\overline{\rho }}(\vec {k}) \ . \end{aligned}$$

Thus, the projected ee interaction potential operator can be rewritten in 2D Fourier space as:

14 $$\begin{aligned} \hat{{\overline{V}}}_{een}=\frac{1}{2} \sum _{\vec {k}} V_{n}(\vec {k}) \, {\overline{\rho }}(-\vec {k}) \, {\overline{\rho }}(\vec {k}) \ , \end{aligned}$$

where $V_{n}(\vec {k})=F_n(k)^2 \, v(\vec {k})$ represents the fully *n*-th LL projected effective interaction potential between electrons and $v(\vec {k})$ is the 2D Fourier transform of the standard Coulomb potential. It is well known that projection onto the *n*-th LL introduces highly non-trivial physics^27^. The key idea of our approach is to assume a uniform circular Fermi liquid state made up of fermions with density operator ${\overline{\rho }}(\vec {k})$ and investigate possible instabilities induced by the renormalized interactions. Such a renormalized interaction is represented by $V_{n}(\vec {k})$ which in real space would be given by $V_n(\vec {r}) = \int d^2k/(2 \, \pi )^2 \, e^{-i \, \vec {k} \,\vec {r}}\, V_n(\vec {k})$.

**Figure 1:**
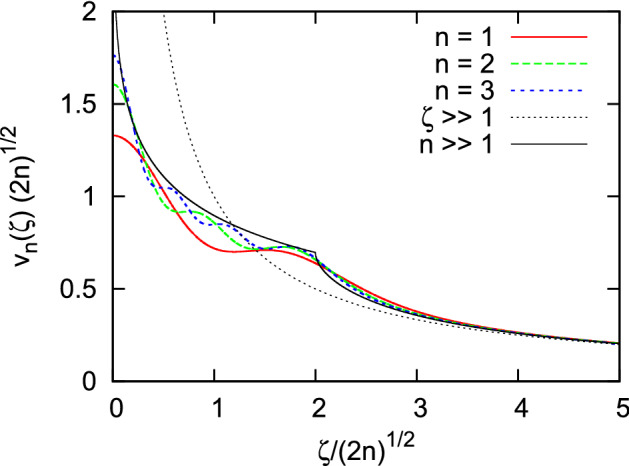
Interaction potentials calculated from Eq. (25) alongside the large-*n* [Eq. (26)] and large-$\zeta$ (Coulomb) asymptotes.

There are two consequences of the projection onto the half-filled Landau level implicit in the quantity given by Eq. (14). Firstly, the effective interaction potential, $V_n(\vec {r})$ is heavily renormalized when compared to the Coulomb interaction. Secondly, the projected density operators, themselves, have a non-trivial algebra. Here, we wish to investigate whether the first of these two features may be sufficient to produce a PI instability in a putative Fermi liquid state. In line with this view, we assume that new fermion creation and annihilation operators $\psi ^{\dagger },\psi$ can be introduced in such a way that the projected densities can be written as ${\bar{\rho }}(\vec {q})=\sum _{\vec {k}}\psi (\vec {k})^{\dagger }\psi (\vec {k}+\vec {q})$ (a form that implies standard operator algebra). Here $\psi ^{\dagger }(\vec {k})$ creates a fermion in a plane wave state. One can then apply the mean-field theory of a PI in a 3D continuum^20^ (which has been recently generalised to a 2D scenario^28,29^). It starts with a Hartree-Fock ansatz for the ground state,

15 $$\begin{aligned} \left| {\varepsilon _{\vec {k}}}\right. \rangle = \prod _{\vec {k}} \left[ \Theta \left( \varepsilon _{\vec {k}} \right) +\Theta \left( -\varepsilon _{\vec {k}} \right) \psi ^\dag (\vec {k}) \right] \left| {0}\right. \rangle , \end{aligned}$$

where $\left| {0}\right. \rangle$ is the vacuum. Note that this ansatz is a homogeneous, itinerant state with an arbitrary dispersion relation, $\varepsilon _{\vec {k}}$. For the Hamiltonian in Eq. (14), this Slater determinant of plane waves affords a rudimentary description of the re-emergence of itinerancy when the kinetic energy has been completely quenched. The expectation value of the energy, $E[\varepsilon _{\vec {k}}] = \langle \left. {\varepsilon _{\vec {k}}}\right| \hat{{\bar{V}}}_{een} \left| {\varepsilon _{\vec {k}}}\right. \rangle$ is:

16 $$\begin{aligned} E[\varepsilon _{\vec {k}}] = N^* V_{n}\left( 0\right) +\frac{1}{2}\sum _{\vec {k},\vec {k}'} \left[ {\bar{V}}_n-V_{n}\left( \vec {k}-\vec {k}'\right) \right] n\left( \vec {k}\right) n\left( \vec {k}'\right) \ , \end{aligned}$$

where $n (\vec {k}) \equiv \langle \left. {\varepsilon _{\vec {k}}}\right| \psi (\vec {k})^{\dagger }\psi (\vec {k}) \left| {\varepsilon _{\vec {k}}}\right. \rangle$ is the occupation number of the plane wave state with wave vector $\vec {k}$, which equals $\Theta \left( -\varepsilon _{\vec {k}}\right)$ in accordance with our *ansatz* and ${\bar{V}}_n \equiv \int d^2r V_n(\vec {r})$. On the other hand, the quantity, $V_n(0)$ in the above expression denotes the effective interaction potential in real space evaluated at zero distance. Note that $V_n(0)$ is finite for our projected interactions as can be seen from Fig. 1.

The functional form of $\varepsilon _{\vec {k}}$ is our variational parameter. It determines which plane wave states are occupied by way of the relation, $\varepsilon _{\vec {k}} \le 0$ and is found by minimization of the functional, $E[\varepsilon _{\vec {k}}]$. The quantity, $\varepsilon _{\vec {k}}$ can be regarded as a mean field and $E[\varepsilon _{\vec {k}}]$ as the thermodynamic potential that is minimized by its equilibrium configuration. Indeed Eq. (16) is a particular case of the Hartree-Fock thermodynamic potential obtained for generic interacting many-fermion Hamiltonians in 3D^20^ as well as in 2D^29^. Note that the work by Quintanilla and Schofield^20^, in particular, shows many details of how the functional, $E[\varepsilon _{\vec {k}}]$ can be obtained as the low-temperature limit of the Hartree-Fock Helmholtz free energy. The minimization yields the self-consistency equation:

17 $$\begin{aligned} \varepsilon _{\vec {k}}= V_n(0) +\sum _{\vec {k}'} \left[ {\bar{V}}_n- V_{n}\left( \vec {k}-\vec {k}^{\prime } \right) \right] n\left( \vec {k}'\right) \ . \end{aligned}$$

Once solved, the self-consistency equation determines the shape of the Fermi surface through $\varepsilon _{\vec {k}}=0$. PI-s are point group symmetry-breaking instabilities of the shape of the Fermi surface. Consider an arbitrary, but infinitesimal variation of the occupation numbers, $n (\vec {k}) \rightarrow n (\vec {k}) +\delta n (\vec {k})$. The corresponding change in the energy, $E \rightarrow E+\delta E$, can be interpreted as the energy of a Landau quasiparticle and is given by the standard expression:

18 $$\begin{aligned} \delta E= \sum _{\vec {k}}\xi _{\vec {k}}\delta n\left( \vec {k}\right) +\frac{1}{2}\sum _{\vec {k},\vec {k}'}f\left( \vec {k},\vec {k}'\right) \delta n\left( \vec {k}\right) \delta n\left( \vec {k}'\right) . \end{aligned}$$

This expression can be obtained straightforwardly by variation of Eq. (16). The quasi-particle dispersion turns out to be identical to our mean field $\xi (\vec {k}) = \varepsilon (\vec {k})$ [given in Eq. (17)] and the Landau interaction function is^29^:

19 $$\begin{aligned} f\left( \vec {k},\vec {k}'\right) ={\bar{V}}_n-V_{n}\left( \vec {k}-\vec {k}'\right) . \end{aligned}$$

Note that, unlike earlier work^28,29^, there is no ‘bare’ contribution to the quasi-particle dispersion - it all comes from interactions.

To find an instability equation, it is natural to split, $\varepsilon _{\vec {k}}$ in two parts: one that preserves the continuous rotational symmetry of the plane and another one that may break it. Thus, we write^29^:

20 $$\begin{aligned} \varepsilon _{\vec {k}} = \varepsilon _{0}(|{\vec {k}}|)-\Lambda _l(|\vec {k}|)\cos (l \, \theta _{\vec {k}}) \ , \end{aligned}$$

where $\varepsilon _{0}(|{\vec {k}}|)$ is the symmetric component of the dispersion relation and $l=1,2,3,\ldots$ determines the symmetry of the instability. The condition of instability towards a small deformation of the Fermi surface is (ignoring the possibility of a first-order phase transition)^28,29^ is:

21 $$\begin{aligned} V_l\ge & {} \frac{4\pi \hbar v_F^0}{k_F^0} \ , \end{aligned}$$

where

22 $$\begin{aligned} V_l = 4\pi \int _0^{\infty }dr ~ r \, V_n(r) \, J_l(k_F^0 \, r)^2 \ , \end{aligned}$$

measures the strength of the ee interaction in the channel with angular momentum, $l=1,2,3,\ldots$ Note that, in the present case, $V_{n}(\vec {r}) \equiv V_{n}(r)$, where $r=|\vec {r}| \ge 0$ is the magnitude of the separation vector.

This is equivalent, within our *ansatz*, to the classic^13^ PI criterion, $F_l = -2$, where the Landau parameter $F_l$ is the $l^{\textrm{th}}$ coefficient of the partial wave decomposition of the Landau interaction function, $f(\vec {k},\vec {k}^{\prime })$^29^, evaluated at the (undistorted) Fermi surface. This describes a divergence of the susceptibility, $\chi _l \sim 1/\left( 2+F_l\right)$ towards Fermi surface deformations of a symmetry given by *l*. For $l=1$, the PI corresponds to a rigid displacement of the Fermi surface in reciprocal space, without change of either shape or volume. This can never lead to a lowering of the energy in a Galilean-invariant system and, therefore, this instability cannot take place^20,29,30^. This also follows from the more explicit form of the instability equation derived below, see Eq. (24). On the other hand for $l=2,3,4\ldots$ we could have PI-s corresponding to deformations of the Fermi surface possessing, respectively, *d*-wave, *f*-wave, *g*-wave.... symmetry.

The Fermi velocity in Eq. (21) is given by^29^:

23 $$\begin{aligned} v_F^0 = \frac{k_F^0}{4\pi \hbar } V_1, \end{aligned}$$

where we take into account the infinite bare mass $m_e \rightarrow \infty$ of our Hamiltonian [see Eq. (14)] (in the sense that the kinetic energy is frozen and, as a result, plays no role). Substituting this into Eq. (21) yields:

24 $$\begin{aligned} V_l-V_1 \ge 0 \ . \end{aligned}$$

The effective interaction can be written as:

25 $$\begin{aligned} V_n(r)=\frac{e^2}{4\pi \epsilon } \int _{0}^{\infty } dk \, J_0(k \, r) \left[ L_n\left( \frac{k^2 \, l_0^2}{2} \right) \right] ^2 \exp \left( -\frac{k^2 \, l_0^2}{2} \right) \ , \end{aligned}$$

where $J_l(x)$ are Bessel functions and we take $n=1,2,\ldots$ We can write $V_n(r)=(e^2/4\pi \epsilon l_0) \, v_n(\zeta )$ where $\zeta =r/l_0$ is the natural dimensionless distance. We calculated $v_n(\zeta )$ exactly for several *n* (we do not display such expressions for the sake of brevity). The results are shown in Fig. 1, where we also plot an asymptotic expression for the effective interaction obtained in the limit of high LL-s^10^:

26 $$\begin{aligned} v_n\left( \zeta \right) \sim \frac{1}{\zeta } \frac{4}{\pi ^2} \, \Re \left[ K \left( \frac{1}{2}-\sqrt{\frac{1}{4}-\frac{2n}{\zeta ^2}} \right) ^2 \right] \ \ ; \ \ n \gg 1 \ , \end{aligned}$$

where *K*(*x*) is a complete elliptic integral of the first kind.

All interaction potentials tend symptotically to the Coulomb interaction, $v_n(\zeta ) \sim {1}/{\zeta }$ for large $\zeta$. This leads to infrared divergences in the theory whose regulation is discussed below. On the other hand, the presence of a length scale in the problem (the magnetic length) is apparent at shorter distances where one notices that as *n* increases an increasingly sharp kink develops at the specific distance $r_n = \zeta _n l_0$ where

27 $$\begin{aligned} \zeta _n \equiv 2\sqrt{2n} \ . \end{aligned}$$

Note in particular that the ultraviolet divergence of the Coulomb interaction at short distance has been suppressed. Interestingly, a sharp feature in the interaction potential at a finite distance $r_n$ suggests the possibility of a PI provided the dimensionless parameter $r_n k_F$ is large enough^20^. Since $r_n k_F = \zeta _n$, Eq. (27) implies an increased tendency towards a PI in high LL-s.

Note that since $v_{n}\left( \zeta \right) \sim 1/\zeta$ for $\zeta \gg 1$ the RHS of Eq. (22) diverges. This leads to diverging Landau parameters, $F_l \propto V_{l}$^29^ and Fermi velocity $v_{F}^{0} \propto V_1$. These infrared divergences can be regulated by introducing a cutoff $\zeta _c$ in the ee interaction potential. The actual values of $v_F$ and $F_l$ depend on the actual value and form of the cutoff which in turn depends on *extrinsic* features of the system such as sample size or device configuration. However, as long as the cutoff distance is long enough, the instability condition does not depend on these parameters. It is such *intrinsic* tendency to a PI that we wish to probe further. To do so let us rewrite the instability condition in Eq. (24) in a more explicit form:

28 $$\begin{aligned} I(n,l) \equiv \int _0^{\infty }d\zeta \, \zeta \, v_n(\zeta ) \, \left[ J_l(\zeta )^2-J_1(\zeta )^2 \right] \ge 0 \ . \end{aligned}$$

Clearly, if $I(n,l) < 0$, a PI does not occur. However, as *I*(*n*, *l*) becomes less negative, the susceptibility to a PI with angular momentum quantum number *l* increases until it diverges at $I(n,l)=0$.

**Figure 2:**
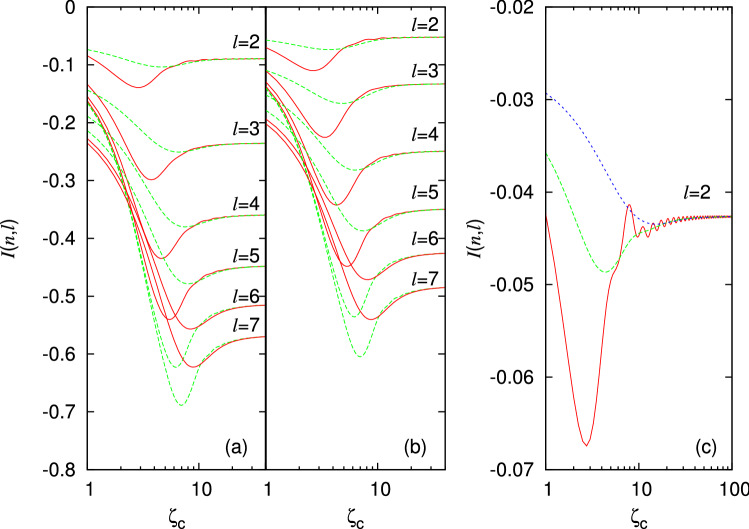
Dependence of *I*(*n*, *l*) in Eq. (28) on the cutoff $\zeta _{c}$ for $w=1$ (red solid curves), 2 (green long dash) and 4 (blue short dash) for (**a**) $n=1$; (**b**) $n=2$; and (**c**) $n=10$ (note the different scales on the third plot). We employed the asymptotic expression in Eq. (26) to plot (**c**). The values of angular momentum *l* are as indicated.

**Figure 3:**
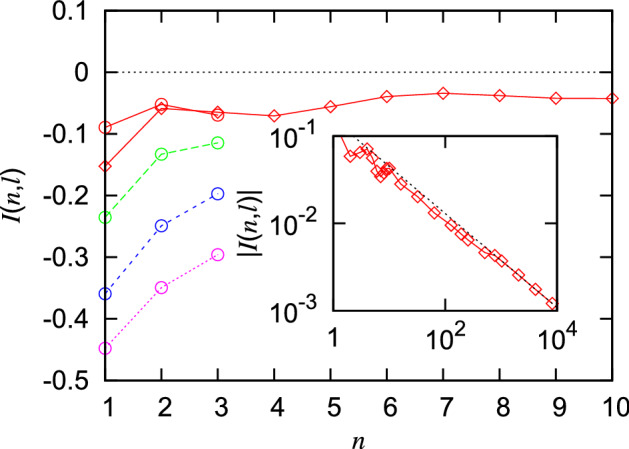
Dependence of the integral *I*(*n*, *l*) in Eq. (28), evaluated numerically, on *n*. The angular momenta of the instabilities are $l=2,3,4 \text{ and } 5$ (solid, long-dashed, short-dashed and dotted lines, respectively). Circles represent values obtained using the effective potentials, $v_n(\zeta )$ for $n=1, 2$ and 3. Diamonds were obtained using the asymptotic formula in Eq. (26). The inset shows the behaviour at very large *n* for the $l=2$ case. The straight line in the inset is a fit of the form $I(n,l) = A_l/n^{B_l}$ to the numerical data for $n \ge 800$. The fitting parameters are $A_2 \approx 0.15$ and $B_2 \approx 0.54$.

The following replacement:

29 $$\begin{aligned} v_{n}\left( \zeta \right) \rightarrow f\left( \frac{\zeta -\zeta _{c}}{w}\right) v_{n}\left( \zeta \right) , \end{aligned}$$

with $f\left( x\right) =\frac{1}{e^{x}+1}$ provides a mathematically convenient way of introducing a cutoff in the form of a smoothed-out step function of finite width *w*. Fig. 2 shows the dependence of *I*(*n*, *l*) on the cutoff $\zeta _c$. For sufficiently large $\zeta _c$ the integral is independent of $\zeta _c$ as well as of the width *w* of the cutoff. Taking the limit $\zeta _c \rightarrow \infty$ represents a convenient way to estimate the *intrinsic* tendency of the system to a PI which is independent of the form and value of the cutoff. Physically, such a cutoff may correspond to, for example, the finite thickness of the device. The crucial point is that the value of the integral *I*(*n*, *l*) in Eq. (28) is an *intrinsic* feature of the system independent of the form, size and mechanism of the cutoff as long as $\zeta _{c}$ is much larger than the average separation between particles $\sim k_{F}^{-1}=l_{0},$ and $\zeta _{c}\gg \zeta _n$ where $\zeta _n$ is the distance where the kink of the effective potential in Fig. 1 appears. The dependence of the converged values of *I*(*n*, *l*) on *n* and *l* is shown in Fig. 3.

We note that the PI condition is never met for $\nu =5/2,\,9/2$ and 13/2. The susceptibility to a PI has a non-trivial dependence on *n* with a maximum for $l=2$ at $n=2$, while it monotonically increases for $l>2$ up to $n=3$. Also note that the system seems most susceptible to a PI in the $l=2$ channel. From these results we conclude that the system never has an intrinsic PI i.e. *I*(*n*, 2) is always $<0$. On the other hand, for large *n* we have $I(n,2) \propto 1/n^{0.54}$, which implies that the half-filled level is asymptotically unstable to a PI in the $n \rightarrow \infty$ limit. The strict $n \rightarrow \infty$ limit corresponds to zero magnetic field, where focusing on an isolated, half-filled LL is not justified. This result must, therefore, be interpreted as an enhanced tendency towards a PI as *n* is increased.

## Conclusions

We have seen that an anisotropic interaction potential between electrons can lead to the stabilization of anisotropic phases for the case of Fermi liquid states of electrons in the quantum Hall regime^31^. However, the current effort is different from such a perspective—we want to investigate how isotropic interaction potentials (with peculiar features) may drive a Fermi liquid system towards anisotropic phases. To this effect, we appeal to the PI mechanism to argue the possibility that a Fermi liquid state of electrons in a half-filled LL may enter a “nematic” phase that breaks the rotational symmetry but preserves the translational one^32^.

The main objective of this work is to investigate whether a compressible circular Fermi liquid state in half-filled high LL-s undergoes a phase transition to a non-circular anisotropic nematic phase by means of a PI transition. We use the Hamiltonian theory approach^26^ to derive a properly LL projected effective ee interaction from which itinerancy emerges at the mean-field level. We look for deformations of the Fermi surface by testing for an *intrinsic* divergence of the corresponding susceptibility. We find that the susceptibility towards a PI is increasingly large as we move to high LL-s and diverges in the $n \rightarrow \infty$ limit. The increased tendency towards a PI is a direct consequence of the length scale $r_n \sim \sqrt{2 \, n} \, l_0$ present in the effective interaction. As is well known, there is also a tendency towards stripe formation^7,8^ with whom the PI competes. In real systems, there is a second length scale, $r_c$, responsible for cutting off infrared diveregences associated with the long-range nature of the Coulomb interaction. Evidently $r_n$ and $r_c$ would become comparable for large values of *n*. The intrinsic susceptibility to a PI is already quite high in this regime.

This means that, even minor intrinsic and/or extrinsic effects (sample thickness, device configuration, etc.) associated with this second length scale may trigger such an instability. The simplest example of a trigger for such an instability that we can think of may involve a small mass anisotropy of the electrons. A 2D system of electrons with anisotropic band mass $(m_x \ne m_y)$ interacting with an isotropic Coulomb interaction potential can be effectively mapped to a 2D system of charged particles with isotropic mass and an anisotropic Coulomb interaction, $v_{\gamma }(\vec {r})=k_e \, e^2/\sqrt{x^2/\gamma ^2+\gamma ^2 \, y^2}$ where $k_e$ is Coulomb’s electric constant, $\vec {r}=(x,y)$ is the 2D vector that separates the positions between a pair of electrons and $\gamma ^2=\sqrt{m_x/m_y}$ is a parameter that depends on the mass anisotropy^33^. This kind of internal anisotropy may trigger an instability in Fermi liquid states since an effective mass anisotropy, at the least, has a tendency to stimulate an elliptical deformation of the Fermi surface^34^. A calculation of the critical value of *n* at which this transition might take place is beyond the scope of the present analysis. It would require taking device configuration into account (finite-thickness effects, etc.) as well as comparing the energy of any Fermi liquid state to those obtained for stripe configurations. In fact, since our preliminary results were made available in preprint form^35^, other relevant research has appeared^36^ accounting for the subtle role played by finite layer thickness effects on bringing a quantum Hall Fermi liquid closer to a PI transition.

## Data Availability

The data are available upon request at J.Quintanilla@kent.ac.uk.

## References

[1] Klitzing KV, Dorda G, Pepper M (1980). New method for high accuracy determination of the fine structure constant based on quantized Hall resistance. Phys. Rev. Lett..

[2] Tsui DC, Stormer HL, Gossard AC (1982). Two dimensional magnetotransport in the extreme quantum limit. Phys. Rev. Lett..

[3] Laughlin RB (1983). Anomalous quantum Hall effect: An incompressible quantum fluid with fractionally charged quasiparticles. Phys. Rev. Lett..

[4] Jain JK (1989). Composite-fermion approach for the fractional quantum Hall effect. Phys. Rev. Lett..

[5] Wang Z, Mandal I, Chung SB, Chakravarty S (2014). Pairing in half-filled Landau level. Ann. Phys. (NY).

[6] Lilly MP, Cooper KB, Eisenstein JP, Pfeiffer LN, West KW (1999). Evidence for an anisotropic state of two-dimensional electron in high Landau levels. Phys. Rev. Lett..

[7] Fogler MM, Koulakov AA, Shklovskii BI (1996). Ground state of a two-dimensional electron liquid in a weak magnetic field. Phys. Rev. B.

[8] Moessner R, Chalker JT (1996). Exact results for interacting electrons in high Landau levels. Phys. Rev. B.

[9] Shibata N, Yoshioka D (2001). Ground-state phase diagram of 2D electrons in a high Landau level: A density-matrix renormalization group study. Phys. Rev. Lett..

[10] Goerbig MO, Lederer P, Morais Smith C (2004). Competition between quantum-liquid and electron-solid phases in intermediate Landau levels. Phys. Rev. B.

[11] Fradkin E, Kivelson SA (1999). Liquid-crystal phases of quantum Hall systems. Phys. Rev. B.

[12] Fradkin E, Kivelson SA, Oganesyan V (2007). Electron nematic phase in a transition metal oxide. Science.

[13] Pomeranchuk II (1958). On the stability of a Fermi liquid. JETP.

[14] Oganesyan V, Kivelson SA, Fradkin E (2001). Quantum theory of a nematic Fermi fluid. Phys. Rev. B.

[15] Doan QM, Manousakis E (2007). Quantum nematic as ground state of a two-dimensional electron gas in a magnetic field. Phys. Rev. B.

[16] Ciftja O, Wexler C (2002). Fermi hypernetted-chain study of half-filled Landau levels with broken rotational symmetry. Phys. Rev. B.

[17] Wexler C, Ciftja O (2006). Novel liquid crystalline phases in quantum Hall systems. Int. J. Mod. Phys. B.

[18] Ciftja O (2020). Detailed solution of the problem of Landau states in a symmetric gauge. Eur. J. Phys..

[19] Ciftja O, Wexler C (2003). Monte Carlo simulation method for Laughlin-like states in a disk geometry. Phys. Rev. B.

[20] Quintanilla J, Schofield AJ (2006). Pomeranchuk and topological Fermi surface instabilities from central interactions. Phys. Rev. B.

[21] Halperin BI, Lee PA, Read N (1993). Theory of the half-filled Landau level. Phys. Rev. B.

[22] Rezayi E, Read N (1994). Fermi-liquid-like state in a half-filled Landau level. Phys. Rev. Lett..

[23] Ciftja O (2006). Monte Carlo study of Bose Laughlin wave function for filling factors 1/2, 1/4 and 1/6. Europhys. Lett..

[24] Girvin SM, Jach T (1984). Formalism for the quantum Hall effect: Hilbert space of analytic functions. Phys. Rev. B.

[25] MacDonald AH (1984). Laughlin states in higher Landau levels. Phys. Rev. B.

[26] Murthy G, Shankar R (2003). Hamiltonian theories of the fractional quantum Hall effect. Rev. Mod. Phys..

[27] MacDonald AH, Girvin SM (1986). Collective excitations of fractional Hall states and Wigner crystallization in higher Landau levels. Phys. Rev. B.

[28] Quintanilla J, Hooley C, Powell BJ, Schofield AJ, Haque M (2008). Pomeranchuk instability: Symmetry-breaking and experimental signatures. Phys. B.

[29] Quintanilla J, Haque M, Schofield AJ (2008). Symmetry-breaking Fermi surface deformations from central interactions in two dimensions. Phys. Rev. B.

[30] Wölfle P, Rosch A (2007). Fermi liquid near a quantum critical point. J. Low Temp. Phys..

[31] Ciftja O (2017). Anisotropic magnetoresistance and piezoelectric effect in GaAs Hall samples. Phys. Rev. B.

[32] Fradkin E, Kivelson SA, Lawler MJ, Eisenstein JP, Mackenzie AP (2010). Nematic Fermi fluids in condensed matter physics. Annu. Rev. Condens. Matter. Phys..

[33] Ciftja O (2021). Origin of the anisotropic Coulomb interaction potential for a two-dimensional system of charged particles with anisotropic mass. Results Phys..

[34] Ciftja O (2021). Deformation of the Fermi surface of a spinless two-dimensional electron gas in presence of an anisotropic Coulomb interaction potential. Sci. Rep..

[35] Quintanilla, J. & Ciftja, O. Does a Fermi liquid on a half-filled Landau level have Pomeranchuk instabilities?. arXiv:0904.0658v2.

[36] Lee K, Shao J, Kim E-A, Haldane FDM, Rezayi EH (2018). Pomeranchuk instability of composite Fermi liquids. Phys. Rev. Lett..

